# Sleep-wake disorder: A silent health crisis in USA

**DOI:** 10.1016/j.amsu.2022.104727

**Published:** 2022-09-19

**Authors:** Abdullahi Tunde Aborode

**Affiliations:** Mississippi State University, Starkville, USA

Dear Editor,

A variety of detrimental physical and social consequences, including poor academic and job performance, are linked to insufficient sleep. 7 of the top 15 causes of death in the United States, including cardiovascular disease, hematological malignancies, cerebrovascular illness, accidents, diabetes, septicemia, and hypertension, have been associated to decreased sleep duration [1]. The research indicates that the relationship between inadequate sleep and negative outcomes is more direct than the relationship between excessive sleep and negative outcomes, which is driven by underlying chronic health issues rather than the other way around [2]. As a result, the impacts of insufficient sleep seem to be more prominent in the US culture and, given their wide-ranging effects, they pose a serious public health risk. This article discusses some of the most severe effects of insufficient sleep as well as how social and behavioral adjustments may be the most effective way to treat these issues.

According to the CDC, people aged 65 and older were most likely to report unknowingly falling asleep during the day (44.6%), and Black Americans (52.4%) were more likely to experience this issue than Hispanics (41.9%) or Whites (33.4%) [3]. People aged 25 to 34 were also more likely than people aged 65 and older to fall asleep while driving (7.2%), and men were more likely to experience this issue (5.8%) than women (3.5%) [4].

Due to the repeated hourly awakenings throughout the course of the night, people with sleep apnea frequently experience a variety of daytime mental symptoms. These signs include lethargy, diminished attention span, irritability, and particularly diminished short-term memory. According to research, those who suffer from sleep apnea have difficulty turning short-term memories into long-term ones [5]. A crucial step in the creation of memories that takes place while people sleep is consolidating memories, or saving events so that people may access them later [5]. When a condition affects sleep, people struggle to integrate and classify their experiences, which impairs memory development and causes forgetting [6].

Children in a family frequently cause the people they live with to get insufficient rest or sleep [7]. A chronic disease, inadequate sleep is frequently attributed to biological or circadian disruption factors. Reviews of studies investigating potential causes have also revealed that genetic variables may be responsible for up to one-third of cases of inadequate sleep [6,7]. Lack of sleep is linked to increased soda intake through excessive salt or carbonated beverage drinking as well as less regular vegetable consumption [8].

Stress of all kinds also plays a role. Uncertainty over housing and food are psychological stressors linked to poor sleep [9,10]. Only social and behavioral health indicators contribute to the explosion of inadequate sleep [11]. Aside from passive smoking, active smoking, and secondhand smoke exposure, inadequate sleep has also been linked [12]. Insufficient sleep and behavioral or emotional issues have been found to have reciprocal and mutually enabling impacts in an increasing number of studies. It is well recognized, for example, that mood disorders are commonly accompanied by significant sleep issues, and that the issues caused by inadequate sleep, such as poor impulse control, a short attention span, and memory problems, may make an already present mood disorder worse [13].

Adolescents who have chronic sleep deprivation, accompanying drowsiness, and daytime impairments face real obstacles to success in school, good health (such as depression and an increased risk of obesity), and safety (such as traffic accidents) [13]. Sleep problems can arise during either sleep initiation or sleep maintenance due to emotional stimulation and pain. Even later bedtimes and sleep cycles that are especially out of sync with daily rhythms might be caused by behavioral disorders and family strife [12]. Furthermore, numerous studies have demonstrated that getting little sleep makes it more likely that workplace mistakes and accidents will occur during the day [12,13].

In the greater scheme of life's challenges, the general public frequently downplays the importance of sleep deprivation and may have a more general attitude that “not getting enough sleep” occupies a relatively low rung on the climbing frame of personal health issues. As a result, during medical interviews, patients frequently fail to mention sleep insufficiency. Some senior experts deal with their patients' sleeping patterns deftly and purposefully, but as the research reviewed here demonstrate, the importance of sleep to health status merits special and sincere care.

Setting predictable wake-up times, avoiding using electronics just before bed, and getting enough exercise are all good ways to make sure people get enough sleep [14]. Employers must be made aware of the importance of sleep for general health as well as their need to create a work environment and conditions that do not obstruct an employee's entitlement to a healthy amount of sleep. Brighter workspaces must be planned for and produced by business management. Managers of businesses should make an effort to reduce conflict and the use of electronic gadgets. Additionally, administrators at the schools should think about setting later start times for classes to prevent interfering with kids' crucial sleep needs, which are present during the adolescent years.

The role that businesses can play in preventing work assignments from negatively affecting sleep cycles should be emphasized by policymakers as they support educational initiatives to increase knowledge of the value of sleep. Teenagers who don't get enough sleep are more likely to be overweight because they don't exercise regularly, have depressive symptoms, engage in risky activities like drinking, smoking, and using drugs, and perform poorly in school, according to research [15].
